# A dataset about Spanish young people’s digital skills, use of technology and online platforms

**DOI:** 10.12688/f1000research.128899.1

**Published:** 2023-01-09

**Authors:** Daniel Aranda, Leila Mohammadi, Mireia Montaña Blasco, Elisenda Estanyol, Pedro Fernández-de-Castro

**Affiliations:** 1 Media Studies, Universitat Oberta de Catalunya, Barcelona, Catalunya, 08018, Spain

**Keywords:** Digital literacy competencies, participation, civic engagement, digital technology use, digital skills, ICT in education, youth, technical competencies, informational competencies, critical knowledge.

## Abstract

**Background**: The datafication scenario of the current communicative ecosystem poses a challenge to media and digital literacy, especially in terms of participation and civic and democratic engagement of youth.

**Methods**: For this purpose, through a survey with a representative sample of 600 young people in Spain, between 16 and 18 years old, we observed their level of digital literacy through three variables: technical competencies, informational competencies, and critical knowledge. This dataset also collects information on the reasons why young people use digital technology such as video games, consoles, computers or mobile phones. On the other hand, we also offer information on the types of social networks or applications and the time and types of uses by youngsters of different digital technologies and social media platforms. The survey includes socio-demographic factors such as gender including (male, female, and others).

**Conclusions**: This survey offers researchers relevant data on the digital skills of Spanish youth and on the perceptions of the use of different digital technologies. This paper also reports the main descriptive data that can be expanded by researchers accessing the database.

## Introduction

Young people need skills and competencies to make the most of the benefits of the internet and digital media. This is especially relevant to bridge the socioeconomic and political and cultural participation gap ( Livingstone *et al*., 2021). Thus, the project in which this dataset frames, explores how certain uses of the internet and social media allow young people to position themselves and stand as political actors or performers involved in social, cultural and economic political life. In this sense, the project includes four sections: use of different technological devices, digital literacy competencies, ICT use in formal and informal learning, and the effect of using digital technologies on social life. To include the impact of COVID-19 crisis on digital social education, the project specifies some relevant questions to during and after COVID-19 lockdown.

## Methods

### Ethics

Informed consent was obtained from participants. The questionnaire was sent to a panel of people from 16 to 18 years of age via Dynata data-platform, that complies with all legal matters in relation to data protection and response protocols. Those aged 16 to 18 answered the children themselves but with the consent of the parents, which were present while they answered as the legislation indicates.

Data and participant security and confidentiality were respected following the UNE EN ISO/IEC 27001 standards and the favorable report issued by the Universitat Oberta de Catalunya (UOC) Ethics Committee under file CE22-PR05.

Research design is quantitative and cross-sectional, involving an online survey intended to measure the self-perceived digital competence of young people aged 16 to 18 living in Spain. This age group was chosen because they need specific skills in order to take full advantage of the benefits of the Internet and social media, especially with regard to reducing the gap in their political and cultural participation ( Theben, 2021). The survey was self-administered, i.e., it was completed by the respondents themselves without the presence of an interviewer, between 23 ^rd^ September and 5 ^th^ October 2021.

The survey is based on studies by Van Deursen *et al*. (2016) and Aranda *et al*. (2020), as well as an extension of the Oxford Internet Institute’s (OxIS) WIP Britain 2013, combined with a systematic review of the notion of “digital youth work” ( Fernández-de-Castro *et al*., 2021). It includes a section of socio-demographic factors begins with gender (male/female, also they have been given the open answer option of other if they perceived themselves non-binary), age, place of residence, the population of the city, level of education, etc. Afterwards there are four further sections comprising of 24 questions about the respondents’ self-perceived digital competence. Because the survey asked young people about how they perceive their own digital skills and knowledge, their answers may not necessarily match their actual competence level.

Due to the importance of COVID-19 crisis in these recent years, the survey includes questions related to using digital technologies for formal and informal education during and after COVID-19 lockdown. Education was an important sector highly affected by the COVID-19 crisis because of the rapid change of teaching to online form. In May 2020, 56.6 % of the total number of students have been affected worldwide and schools had been forced to close in 130 countries ( Donohue & Miller, 2020). Spain lockdown started on 15 ^th^ March 2020 until 21 ^st^ June 2020 and with the start of the new school year in September 2020 most schools reopened.

The first section of the survey, digital technology use and lockdown, begins by asking the participants about use of different technological devices (mobile, tablet, computer, game console and smart watch) and the purposes for which they are used (work, information, training, communication, entertainment and political participation) during and after lockdown. This section continues with three questions about comparing the spending time and its quality on online activities during lockdown (between 15 ^th^ March 2020 and 21 ^st^ June 2020) and after lockdown. The answers were given on a 3-point Likert scale 1: less; 2: the same; 3: more.

The second section is about digital competence and asked about technical skills (nine items), informational skills (ten items), and critical digital knowledge (five items), respectively. Answers were given on a 5-point Likert scale: 1: I don’t know what this is or what it means; 2: I know what this is but I don’t know how to do it; 3: I would know how to do this with help; 4: I know how to do this by myself; and 5: I know how to do this and could teach others. The third section dealt with critical knowledge of the digital environment and included five items, also measured on a 5-point Likert scale: 1: Nothing at all; 2: A little bit; 3: An average amount; 4: A fair amount; and 5: A lot.

The third section on ICT use in formal and informal learning asked about self-learning (three items) and nine questions of online formal education which included; training in digital technologies and engagement (four items), online classes and teaching initiatives (five items).

The fourth section is about youth perception and digital technologies, asking the young people to rank five social ability items considering the relevance of digital technologies for them. Then they were asked to rank the effect of using digital technologies on ten social life aspects. Answers were given on a 5 point Likert scale from very negatively (1) to very positively (5).

A principal component analysis (PCA) was carried out on the proposed scales to check their validity and Cronbach’s alpha was used to measure their reliability. Regarding the first section about uses of digital technologies, the analysis showed an acceptable structure for all items Kaiser–Meyer–Olkin (KMO) test results in a mean of 0.738 and the Bartlett test is significant p<0.001). The PCA results offer a structure of a single component that explains 59.5% of the variance. The reliability according to Cronbach’s Alpha coefficient is 0.755.

Regarding the section on technical digital skills, the analysis showed an acceptable structure for all nine items (KMO=0.910; Bartlett’s test significant with p<0.001). The structure comprised two components explaining 64.8% of the total variance (40.1% for the first component and 24.7% for the second); the Cronbach’s alpha coefficient was 0.903 for the first component and 0.773 for the second. For the section on informational digital skills, the analysis showed an acceptable structure for all ten items (KMO=0.955; Bartlett’s test significant with p<0.001). A single component explained 59.9% of the total variance and Cronbach’s alpha was 0.925. For the section on critical digital knowledge, the analysis showed an acceptable structure for all five items (KMO=0.843; Bartlett’s test significant with p<0.001). A single component explained 58% of the total variance and Cronbach’s alpha was 0.819.

## Sample of study

Through a simple random sampling strategy, 600 youth completed the questionnaire, with an average duration of 13 minutes per person. The sample response rate was 62.11%, with a margin of error of 4% for the sample set, with a 95% confidence level (1.96 sigmas) and maximum indeterminate P=Q=50%. Subsequently, stratification was weighted to fine-tune the weights of the interviewees with the population data from the final study universe. The weight coefficient reference data were calculated and using the variables “Nielsen area”, “municipality size”, “gender” and “age” from the last wave of the Spanish General Media Study (EGM).

## Results

The results were processed using IBM SPSS Statistics 24®. We first performed a descriptive statistical analysis of the survey’s Likert scale variables, including calculating means and standard deviations. In what follows, we highlight key data relating to the respondents’ autonomously self-reported skills.

Regarding the purposes that they use different digital technology ( Table 1), the results show that the majority of young people (87.5%) use mobile for entertainment, (59.7%) use computer for information and (58.7%) for work, (46.2%) of them use video game console for entertainment but 50% do not use it at all. More than half of them (59.2%) don’t use tablets, and smart watches (66%).

**Table 1.** Purposes of use of digital technologies.

	Work	Information	Education	Communication	Entertainment	Activism (participation in movements, especially of a political or social nature)	I don’t have one/I don’t use one
Mobile	36.2	68	38.2	77.7	87.5	14.8	1.5
Tablet	13.5	15.2	12.7	8.8	28	2.2	59.2
Computer (laptop or desktop)	58.7	59.7	50.7	36.8	53.2	6.7	9.7
Video game console	1.8	2.5	1.8	5.5	46.2	1.7	49.7
Smartwatch	5	19	4.8	10.8	8.2	2.3	66

With regard to the purposes of using digital platforms, apps or social media, ( Table 2), the results show that the majority of young people (86.7%) use WhatsApp for communication, YouTube (89.7%), Instagram (86.5%), and TikTok (75.2%) for entertainment, while the majority of them don’t use Telegram (60.2%), LinkedIn (80.7), Facebook (66.2%), Snapchat (64.2%), Twitter (46.2%), Twitch (59.7%), and Discord (56%).

**Table 2.** Purposes of using digital platforms, apps or social media.

	Work	Information	Education	Communication	Entertainment	Activism (participation in movements, especially of a political or social type)	I don’t have/I don’t use it
WhatsApp	23	26.3	11.8	86.7	39	5.2	2
Telegram	6.3	12.2	4.7	17.5	17.2	1.8	60.2
LinkedIn	12.5	5.7	2.7	2.8	3	1.2	80.7
YouTube	6.7	46.7	22,7	8.5	89.7	5.2	3.2
Facebook	1.8	11.2	2.8	15.5	20	2.8	66.2
Twitter	1.3	27.5	2.3	22	37.3	6.2	46.2
Instagram	5.8	37.5	6.8	65.8	86.5	9.2	4.7
TikTok	2.8	13.5	2.3	13.3	75.2	4	20.2
Snapchat	0.5	1.7	2	12.7	27	1.5	64.2
Twitch	2.5	3.2	1	3.8	36	0.5	59.7
Discord	4	4.8	2.3	33.3	22.3	1.5	56

Regarding time, 64.3% of the young people claimed that they spent more time as an internet user since the start of the COVID-19 coronavirus pandemic. In what follows, the respondents specified the tendency of their time spent on different activities using the internet during spring 2020 home lockdown. They also asked to compare the quality of each activity ( Table 3). The results demonstrate that the young people spent more or the same amount of time on all the mentioned activities except participating in cultural activities in which (53.8%) of them claimed spending less time during lock down. In respect of quality, the majority of the activities were declared to be of the same quality or better. According to the respondents, activities such as communication with friends, listening to music and watching series had better quality during lockdown. However, they mentioned less quality for studying which required remote school activities during lockdown.

**Table 3.** Comparing the frequency of time and the quality of online activities during the lockdown.

Activity	Amount of time			Quality		
	Less	The same	More	It was of worse quality	It was of equal quality	It was of better quality
Checking current news	12.3	30	57.7	17.7	52.8	29.5
Doing family activities	19.2	34.8	46	17.8	49.5	32.7
Communicating online with family	12.2	34	53.8	14.2	51.8	34
Communicating online with friends	8.8	19.8	71.3	15.3	39.2	45.5
Connecting online with new people	18.3	41.5	40.2	16	55.7	28.3
Studying (required remote school activities)	30.5	29.5	40	45.5	33.7	20.8
Performing adapted after-school activities at home (sports, music, foreign language, etc.)	40	31.5	28.5	39.8	41.5	18.7
Self-taught training (attending non-mandatory talks, watching video tutorials, taking online courses, etc.)	26.5	39	34.5	24.3	52.2	23.5
Playing video games on a computer, console or mobile phone (alone)	13	30.7	56.3	9.5	50.5	40
Playing video games on a computer, console or mobile phone (connected with other players)	15.5	28.7	55.8	12.8	46.2	41
Online gambling	40.8	48.7	10.5	34.2	56.2	9.5
Listening to music	7.3	17.2	75.5	5.3	35.3	59.3
Reading	23	40.8	36.2	16.7	52.5	30.8
Watching series on streaming channels (Netflix, HBO, Movistar Plus+, etc.)	6.5	19.2	74.3	7	34.2	58.8
Activism (participating in movements, especially of a political or social nature)	35.8	50.8	13,3	29.7	59.2	11.2
Participating in cultural activities (concerts, museums, etc.)	53.8	36.7	9.5	37.8	51.7	10.5
Exercising (inside the home)	20.5	28.2	51.3	19.8	43.7	36.5

With regard to technical skills ( Table 4), the results show that 73.4% of young people claim to know how to install/uninstall basic programs and applications without help. Most young people report knowing how to browse the internet and use related services for everyday purposes, with 80.7% saying they can do this without help. Fewer young people appear to use content management platforms to produce multimedia publications, with only 26.6% claiming to know how to do this without help. Meanwhile, 58.4% of the respondents say they know how to record, edit and upload video content to the internet without help. As for sharing and distributing digital multimedia content, 69.7% of the young people surveyed say they can do this without help. A total of 66.6% of young people say they know how to work with others using digital collaboration tools without help. Only 27.8% of young people say they know how to set up digital services and use tools to increase online privacy and anonymity without needing help. In terms of knowing how to read and/or write computer code, only 19.2% say they can do this without help. Similarly, few young people claim to know how to repair and/or service devices without help (25.5%).

**Table 4.** Technical skills of young people.

	Know how to do it	Need help to do it	Don't know how to do it
Install/uninstall basic programs and applications for my needs	73.4	11.1	8.5
Browse the internet and use related services for everyday purposes	80.7	7.6	6.9
Manage content management platforms to produce multimedia publications	26.6	21.3	31.5
Save, edit and upload audiovisual content to digital platforms	58.4	21.3	12.1
Share and distribute multimedia content on networks/platforms/email	69.7	11	11.2
Work with other people using digital collaborative tools	66.6	14.5	11.4
Set up digital services and use tools to increase privacy and anonymity online	27.8	24.8	31
Read and/or write computer codes	19.2	23.8	35.1
Repair and/or maintain devices	25.5	26.8	23.4

In terms of informational skills ( Table 5), 49.4% of young people say they know how to check the reliability and truthfulness of information without help. Among young people, 54% say they know how to classify and filter information to suit their interests without help. A total of 69.9% of young people 69.9% say they are able to find and save information for use when they need it. With respect to social informational skills, 64.4% of the respondents say they know how to display self-control when interacting with others on social media and digital forums so as not to react impulsively. Regarding spotting so-called “trolls” in online discussions, 58.5% of the young people surveyed claim to have this skill, while 47.3% report knowing how to tell when they are interacting with a bot. According to this sample, 68.8% of young people are able to manage the various profiles that make up their digital identity. Meanwhile, 68.2% say they know how to adapt their behavior according to the standards of each platform. Among the young people surveyed, 56.8% report being able to identify their needs and find tools and platforms to fulfill them without help. Less than half of the young people in our sample (45.5%) say they are able to take part in online deliberation and decision-making processes; 17.6% say they know how to do this and could teach others, while 27.9% say they simply know how to do this alone.

**Table 5.** Informational skills of young people.

	Know how to do it	Need help to do it	Don't know how to do it
Check the reliability and veracity of the information I consume	49.4	22.6	17.5
Sort and filter information to fit my interests	54	18.6	18.4
Find and save information to use when you need it	69.9	12.2	11.5
Interact with other people in networks and digital forums with self-control so as not to react impulsively	64.4	11.9	15.1
Identify users who act in an explicitly provocative way	58.5	14	16.7
Distinguish interaction with a bot in digital networks and forums	47.3	16.3	22.5
Manage different profiles of my digital identity (network accounts)	68.8	13	11.5
Adapt my behavior according to the rules of each platform	68.2	10.2	12.1
Identify my needs and find tools and platforms that cover them	56.8	16	16.2
Participate in online deliberation and decision-making processes	45.5	21.8	18.7

In terms of critical knowledge ( Table 6), 22.9% of the respondents say they know a lot or a fair amount about the basic features of digital services. Of the young people surveyed, 33.6% say they know a lot or a fair amount about how technology companies use personal data. Meanwhile, 21.7% of young people say they know a lot or a fair amount about laws dealing with issues related to digital technologies. Only 22.4% say they know a lot or a fair amount about the influence of technology companies on public policy. Finally, 31.9% of young people say they know how the technological devices they use are manufactured.

**Table 6.** Digital knowledge of young people.

	High	Average	Poor
The basic characteristics of the digital services I use	22.9	26.6	41.4
The use that technology companies make of personal data	33.6	29.3	30.7
The laws that deal with issues related to the internet and digital technologies	21.7	28.4	41.5
The influence of technology companies on politics	22.4	26.7	40.2
How the technological devices you use are made	31.9	30.5	28.6

With regard to ICT use in formal and informal learning, the results show that the first three topics for which the young people use the internet to find information are education 60.5%, digital technology 51.2%, and job-related information 48.9%. Regarding the use of ICT to acquire or improve skills, the first four fields reported by young people are video games 54.6%, sport 51.6%, cooking 43.2% and fashion & beauty 42,4%. In respect of self-directed learning, they have been asked to choose the first three things to find information. The results show that first watching online videos (62.7%), then browsing online resources such as books and article 57.7%), and finally asking a family member, or a friend (54.8%).

In reference to specific training in digital technologies in secondary school or university 34.6% said that they attended talks about uses (security, cyberbullying, etc.), 26.6% attended specific training sessions at their school or university, while 28.4% did not receive such training. With respect to class participation rating, the majority, 29.6% of young people, claim a fairly engagement during in person classes while the majority of them, 31.4% said they engage somehow during online learning. Regarding the procedures used in online education by schools, 64.4% of respondents reported that the most used initiative that conducts online learning activities is Emailing study materials with supporting tasks and instructions. On the subject of use and assessment of different online teaching initiatives, 92.7% voted for interactive exercises, 90% Online forms (no assessment) and 89.6% for online games. Then they have been asked to assess their experience in online classes. 39.6% of the young people claim their disagreement to the statement “my teachers stimulate my interest during online classes” while 38.6% of them neither agreed nor disagreed. Similar results have been seen about the second statement “my teachers come well prepared and organized for each online class”, 36.8% disagreed and 33.4% assessed it as average.

On the subject of use and assessment of different online teaching initiatives, 92.7% voted for interactive exercises, 90% online forms (no assessment) and 89.6% for online games. Then they were asked to assess their experience in online classes. 39.6% of the young people disagree with the statement “my teachers stimulate my interest during online classes’, while 38.6% of them neither agreed nor disagreed. Similar results have been seen regarding the second statement, “my teachers come well prepared and organized for each online class”, 36.8% disagreed and 33.4% assessed it as average. The answer to their preference towards synchronous online classes (all participants connected at once) rather than asynchronous (via videos, material or educational resources previously provided by the teacher) were similar three grades of agreement. Finally, the majority of the young people 62.7% evaluated that the outcome of education during the pandemic (in online format) was worse than before.

In relation to perception of young people about digital technologies, the ranking according to how relevant they think digital technologies are for young people was as following: 26.8% rated first the creating a collective identity (e.g., forming groups to share likes, interests or concerns), participating in activities proposed by institutions 21.9%, achieving common goals (e.g., organizing events or activities) 21.2%, making claims and carrying out actions in-person and non-virtual environments 15.3%, and co-designing the activities in which they participate 14.8%.

With respect to the impact of use of digital technologies on young people ( Table 7), the following concepts have been most as ranked neither positive nor negative: psychological well-being (42.7%), Communication with adults (42.3%), individual identity (37%), ability to organize themselves into groups (37.8%), Acceptance of established social norms (42.2%), and decision-making and social autonomy (37.9%). However, the concepts of ability to argue and discuss (39%), socialization among equals (39.7%), and the ability to express themselves as individuals (34.8%), have been most ranked positively.

**Table 7.** The reception of the impact of the use of digital technologies by young people.

	Very negatively	Negatively	Neither negative nor positive	Positively	Very positively
Young people’s psychological well-being	9.2	25.1	42.7	18.3	4.6
Young people’s ability to argue and discuss	4.9	13.6	32.3	39.0	10.2
Socialization among equals	6.2	13.2	26.2	39.7	14.8
Communication with adults	6.1	17.6	42.3	23.7	10.3
Young people’s individual identity	7.3	20.6	37.0	25.3	9.8
Young people’s ability to express themselves as individuals	5.2	16.9	30.9	34.8	12.1
Group membership	6.0	11.6	36.7	34.3	11.3
Young people’s ability to organize themselves into groups	4.7	8.9	37.8	33.0	15.5
Acceptance of established social norms (rules that people in a community must follow to maintain good social harmony)	3.1	16.9	42.2	27.1	10.7
Decision-making and social autonomy (addressing and making decisions on one’s own initiative)	4.0	20.0	37.9	27.7	10.3

## Data Availability

Figshare: A data set about digital literacy competencies among youngsters (16-18) in Spain https://doi.org/10.6084/m9.figshare.21379104.v3 ( Mohammadi *et al*., 2022) This project contains the following underlying data:
• Dataset.sav • Questionnaire.pdf Data are available under the terms of the Creative Commons Attribution 4.0 International license (CC-BY 4.0).
