# Supplementary material for: A dataset about Spanish young people’s digital skills, use of technology and online platforms
Source: F1000Res. 2023 Oct 27;12:26. Originally published 2023 Jan 9. [Version 2] doi: 10.12688/f1000research.128899.2 (PMC10905144; doi:10.12688/f1000research.128899.2)
Supplement: Peer review history

# Reviewer report: Reviewer response for version 2

**DOI:** 10.5256/f1000research.158037.r234443

**Published:** 2024-02-07

**Authors:** Kolawole Francis Ogunbodede

**Affiliations:** 1 The University of Africa Toru-Orua, Yenagoa, Bayelsa State, Nigeria

Based on the information provided, here is a critical review of the article:

 The article presents a dataset on Spanish youth's digital skills, technology use, and online platform use. The strengths of the article include:

- The dataset addresses an important issue - youth digital literacy and technology use. As digital technologies become more integrated into daily life, understanding how youth engage with and utilize these technologies is crucial.
- The sample size is substantial at 600 Spanish youth aged 16-18, allowing for more robust analysis. The sampling also aimed for representation across different regions of Spain.
- The survey instrument covers key aspects of digital literacy including technical skills, information skills, critical knowledge, as well as technology access and use. This provides rich data for analysis.
- The data have been made openly available for other researchers to utilize. This facilitates further scholarship in this area.
- The article is generally well-written and organized, providing a clear overview of the research background, objectives, methodology, results, and conclusions.

However, some limitations should be noted:

- **Literature Review:** While the article touches upon the challenges posed by the datafication scenario and the importance of digital literacy, a more extensive literature review could strengthen the theoretical foundation. Including more references to relevant studies, frameworks, and theories on digital literacy and youth engagement would enhance the scholarly contribution.
- **Discussion Section:** The discussion of results could be more detailed. Providing deeper insights into the implications of the findings, discussing their alignment with existing literature, and suggesting avenues for future research would enrich the discussion section.
- As a self-reported survey, the data reflect youths' perceptions of their skills and technology use rather than objective measures.
- Some survey questions could have been clarified or expanded, for instance questions on activism and social media use for different purposes.
- There is limited discussion of the context of the findings or implications. As a data note this is understandable, but expanded analysis and discussion of implications would make the data more impactful.
- Sampling procedures and representativeness could have been described in more detail. Information on respondent demographics other than age and gender is also lacking.

Overall this data note provides valuable data that can enable further research on youth digital literacy and technology engagement. As digital technologies continue to evolve, updating the data over time could provide important insights into trends. Addressing some of the survey and sampling limitations noted above could also strengthen future iterations. Expanding analysis and discussion of the findings would be beneficial as well.

Are sufficient details of methods and materials provided to allow replication by others?

Yes

Is the rationale for creating the dataset(s) clearly described?

Yes

Are the datasets clearly presented in a useable and accessible format?

Yes

Are the protocols appropriate and is the work technically sound?

Yes

Reviewer Expertise:

librarianship, ICT, Information literacy, etc.

I confirm that I have read this submission and believe that I have an appropriate level of expertise to confirm that it is of an acceptable scientific standard.

# Reviewer report: Reviewer response for version 2

**DOI:** 10.5256/f1000research.158037.r218884

**Published:** 2023-11-10

**Authors:** Pritika Reddy

**Affiliations:** 1 Department of Computing Science and Information Systems, Fiji National University, Nasinu, Central Division, Fiji

All good from my side.

Are sufficient details of methods and materials provided to allow replication by others?

Partly

Is the rationale for creating the dataset(s) clearly described?

Yes

Are the datasets clearly presented in a useable and accessible format?

Yes

Are the protocols appropriate and is the work technically sound?

Partly

Reviewer Expertise:

NA

I confirm that I have read this submission and believe that I have an appropriate level of expertise to confirm that it is of an acceptable scientific standard.

# Reviewer report: Reviewer response for version 1

**DOI:** 10.5256/f1000research.141536.r207955

**Published:** 2023-09-20

**Authors:** Pritika Reddy

**Affiliations:** 1 Department of Computing Science and Information Systems, Fiji National University, Nasinu, Central Division, Fiji

1. The Abstract can improve. Include more details and keep within the word limit for the abstract.
2. The Introduction is very short and doesn’t explain the need for the study. There needs to be description about important aspects on the topic plus the contribution of the study.
3. Literature Review on digital literacy and media literacy is missing. You can refer to the following papers: Reddy *et al.* (2022 ^1^), Reddy *et al.* (2021 ^2^), Reddy *et al.* (2019 ^3^) and Reddy *et al.* (2023 ^4^).
4. Results and Discussion sections look good.
5. Conclusion and Limitations of the study are missing.

Are sufficient details of methods and materials provided to allow replication by others?

Partly

Is the rationale for creating the dataset(s) clearly described?

Yes

Are the datasets clearly presented in a useable and accessible format?

Yes

Are the protocols appropriate and is the work technically sound?

Partly

Reviewer Expertise:

NA

I confirm that I have read this submission and believe that I have an appropriate level of expertise to confirm that it is of an acceptable scientific standard, however I have significant reservations, as outlined above.

## References

[1] : Digital literacy: a review in the South Pacific. *Journal of Computing in Higher Education* .2022;34(1) : 10.1007/s12528-021-09280-4 83-108 10.1007/s12528-021-09280-4

[2] : Contextualized game-based intervention for digital literacy for the Pacific Islands. *Educ Inf Technol (Dordr)* .2021;26(5) : 10.1007/s10639-021-10534-y 5535-5562 10.1007/s10639-021-10534-y 33935574 PMC8065331

[3] : Predicting Media Literacy Level of Secondary School Students in Fiji. *2019 IEEE Asia-Pacific Conference on Computer Science and Data Engineering (CSDE)* .2019; 10.1109/CSDE48274.2019.9162411 1-7 10.1109/CSDE48274.2019.9162411 Reference source

[4] : A digital literacy model to narrow the digital literacy skills gap. *Heliyon* .2023;9(4) : 10.1016/j.heliyon.2023.e14878 e14878 10.1016/j.heliyon.2023.e14878 37025876 PMC10070644

# Response

**Published:** 2023-10-24

**Authors:** Daniel Aranda

**Affiliations:** Open University of Catalonia, Spain

Estemeed reviewer,

 First of all, thank you very much for taking the time to read and comment our work.

**1. The Abstract can improve. Include more details and keep within the word limit for the abstract.**

 Following your advice, we have extended the abstract using the remaining space available without surpassing the word limit. We have developed the background trying to precise the rationale for this dataset and we have rewritten the method part in a more systematic way adding more details of the procedure.

**2. The Introduction is very short and doesn’t explain the need for the study. There needs to be description about important aspects on the topic plus the contribution of the study.**

 In this section, taking into account your comment, we added a paragraph in order to provide a better context of the research and make more explicit the reasons that fostered our approach. In particular, we reference the work of Middaugh & Kane (2013) New media as a tool for civic learning, Comunicar, Vol.20, No. 40, pp. 99–107. https://doi.org/10.3916/C40-2013-02-10 given that for us is one of the most notable works in our line of research, that is, the civic potential of digital media specifically for young people.

**3. Literature Review on digital literacy and media literacy is missing. You can refer to the following papers: Reddy et al. (2022), Reddy et al. (2021), Reddy et al. (2019) and Reddy et al. (2023).**

 In this point, we decided not to extend the text in terms of a literature review given that, following the guidelines of the F1000 Research journal for Data Notes, this kind of paper does not need that kind of development, although we agree on the need of a literature review in the case of a regular paper. Anyway, we really appreciate the references provided because they are very insightful in relation to our line of research and we will make use of them in following works.

**4. Results and Discussion sections look good.**

 We are thankful for the positive feedback regarding these sections.

**5. Conclusion and Limitations of the study are missing.**

 In the same sense that we discuss in point 3, the guidelines of the F1000 Research journal for Data Notes does not indicate the need for a Conclusion an Limitations section. We invite you to check one article derived from this dataset in which we expose that kind of reflections: Estanyol, E., Montaña, M., Fernández-de-Castro, P., Aranda, D., & Mohammadi, L. (2023). Digital competence among young people in Spain: A gender divide analysis. [Competencias digitales de la juventud en España: Un análisis de la brecha de género]. Comunicar, 74, 113-123. https://doi.org/10.3916/C74-2023-09

 Thank you very much again and we are in touch for further communications.

 Greetings.

# Reviewer report: Reviewer response for version 1

**DOI:** 10.5256/f1000research.141536.r159561

**Published:** 2023-01-27

**Authors:** Mònica Figueras-Maz, Mittzy Arciniega-Caceres

**Affiliations:** 1 Communication Department, Pompeu Fabra University, Barcelona, Spain

The paper focuses on the exploitation of an interesting big sample and a very relevant target; however, it needs a deep revision to go deeper into the analysis and make evident the contribution of the study, moreover present the data. In addition, more detail is needed in the data and ethic isues.

**General comments:**

- In the abstract, the authors use two different concepts: the level of digital literacy and the perceptions of the use of different digital technologies; those are two different approaches. What are the authors exactly measuring?
- The reviewers miss a more solid introduction and a theoretical framework, we suggest reviewing Ferrés and Mateus's studies.
- We also find that the paper lacks conclusions and discussions that go over the exposure of data; it would be interesting to know what is the researchers' position, contribution, and possible recommendations.
- A gender perspective focus could be important, for example, comparing uses and competencies by gender. In addition, other variables could be compared as age, and kind of school (private, public, etc.).

**Sample:**

- Important to describe how researchers guarantee the pluralism of the sample (different socio-economic areas, etc.). Especially regarding access to technology.
- In general, we find more information is needed on sampling and how it is distributed by independent variables. How was the questionnaire distributed, etc.
- Include variables of schools ownership (public, private or semi-private schools).

**Ethics:**

 It would be interesting to get more details about the consent form obtention procedure, as well as general ethics issues. For example, it is said that “those aged 16 to 18 answered the children themselves but with the consent of the parents, which were present while they answered as the legislation indicates”.  How could the authors be sure of this statement?

Are sufficient details of methods and materials provided to allow replication by others?

Partly

Is the rationale for creating the dataset(s) clearly described?

Yes

Are the datasets clearly presented in a useable and accessible format?

Partly

Are the protocols appropriate and is the work technically sound?

Partly

Reviewer Expertise:

NA

We confirm that we have read this submission and believe that we have an appropriate level of expertise to confirm that it is of an acceptable scientific standard, however we have significant reservations, as outlined above.

# Response

**Published:** 2023-03-03

**Authors:** Daniel Aranda

**Affiliations:** Open University of Catalonia, Spain

We appreciate the review, the time spent and the comments.

**Reviewers:**

**The paper focuses on the exploitation of an interesting big sample and a very relevant target; however, it needs a deep revision to go deeper into the analysis and make evident the contribution of the study, moreover present the data. In addition, more detail is needed in the data and ethic isues.**

**Response:**

 As stated in the guidelines a Data Notes are brief descriptions of scientific datasets that promote the potential reuse of research data and include details of why and how the data were created; they do not include any analyses or conclusions. 

 More details on data and also Ethic issues are answered below.

**Reviewers:**

**General comments: I** **n the abstract, the authors use two different concepts: the level of digital literacy and the perceptions of the use of different digital technologies; those are two different approaches. What are the authors exactly measuring?**

**Response:**

 In the abstract, we specifically refer to the fact that the survey reflects on the one hand the competences of young people and on the other hand the perceptions of the use of different digital media or digital technology. These two aspects are effectively different as the reviewers point out and each aspect (Perceptions of use and competencies) has distinct spaces in the questionnaire.

 The perception of the use of digital technologies has been studied in several academic disciplines. According to Davis (1989), Technology Acceptance Theory (TAM) is a theoretical model that explains people’s adoption and use of technology. TAM maintains that perception of the use of a technology is influenced by perception of utility and ease of use. The more useful and user-friendly a technology is, the more likely it is that a person will adopt and use it regularly.

- Davis, F. D. (1989). Perceived usefulness, perceived ease of use, and user acceptance of information technology. MIS quarterly, 13(3), 319-340.

On the other hand, Digital literacy refers to the ability to use digital technologies, such as computers, smartphones, and the internet, to find, evaluate, create, and communicate information effectively. It encompasses a range of skills, including the ability to use digital tools to access and analyze information, collaborate with others, and solve problems.

 Van Deursen and van Dijk (2015) found that digital skills were positively associated with social and economic outcomes, such as employment status, income, and civic engagement. The study also found that individuals with lower levels of digital skills were more likely to experience social and economic disadvantages.

- Van Deursen, A. J. A. M., & Van Dijk, J. A. G. M. (2015). The digital divides shifts to differences in usage. New Media & Society, 17(7), 1-18.

Thus, the second section of the survey is about digital competence: technical skills (nine items), informational skills (ten items), and critical digital knowledge (five items), respectively. 

 The fourth section is about youth perception of digital technologies, asking the young people to rank five social ability items considering the relevance of digital technologies for them. 

**Reviewers:**

**The reviewers miss a more solid introduction and a theoretical framework, we suggest reviewing Ferrés and Mateus's studies.**

**Response:**

 There is no theoretical framework in the data note because it is not required as stated in the guidelines. What reviewers call the theoretical framework makes reference to methodology, a detailed account of the protocol used to generate the dataset.

****

**Reviewers:**

**We also find that the paper lacks conclusions and discussions that go over the exposure of data; it would be interesting to know what is the researchers' position, contribution, and possible recommendations.**

**Response:**

 Data Notes are brief descriptions of scientific datasets that promote the potential reuse of research data. As stated before, this data notes do not include any analyses or conclusions.

**Reviewers:**

**A gender perspective focus could be important, for example, comparing uses and competencies by gender. In addition, other variables could be compared as age, and kind of school (private, public, etc.).**

**Response:**

 Although data note do not include any analyses or conclusions, in relation with gender, readers can see Estanyol, E., Montaña, M., Fernández-de-Castro, P., Aranda, D., & Mohammadi, L. (2023). Digital competence among young people in Spain: A gender divide analysis. [Competencias digitales de la juventud en España: Un análisis de la brecha de género]. Comunicar, 74, 113-123. https://doi.org/10.3916/C74-2023-09

 In this paper we use the notion of digital citizenship, in order to study the gender digital divide as it relates to competence (i.e., skills and knowledge) and the possibility of leveraging said competence to promote civic education grounded in gender equality in the digital environment.

**Reviewers:**

**Sample:**

- **Important to describe how researchers guarantee the pluralism of the sample (different socio-economic areas, etc.). Especially regarding access to technology.**
**  **
- **In general, we find more information is needed on sampling and how it is distributed by independent variables. How was the questionnaire distributed, etc.**

**Response:**

 As stated in the guidelines of Data Notes: for standard protocols that have been published elsewhere, a brief description and reference is sufficient. The detail of the sampling has been published previously in the article "Digital competence among young people in Spain: A gender divide analysis" in the Comunicar Journal.

 Estanyol, E., Montaña, M., Fernández-de-Castro, P., Aranda, D., & Mohammadi, L. (2023). Digital competence among young people in Spain: A gender divide analysis. [Competencias digitales de la juventud en España: Un análisis de la brecha de género]. Comunicar, 74, 113-123. https://doi.org/10.3916/C74-2023-09

 The readers could also have all this  information in the section Data availability: Figshare: A data set about digital literacy competencies among youngsters (16-18) in Spain https://doi.org/10.6084/m9.figshare.21379104.v3 ( Mohammadi et al., 2022).

**Reviewers:**

**Include variables of schools ownership (public, private or semi-private schools).**

**Response:**

 Following the international tradition, we have chosen not to ask for the public or private variable in the survey but as stated by the reviewers this variable could have been interesting to include. Here are some references.

 de Lenne, O., Vandenbosch, L., Eggermont, S., Karsay, K., & Trekels, J. (2020). Picture-perfect lives on social media: A cross-national study on the role of media ideals in adolescent well-being. Media psychology, 23(1), 52-78.

 Jarman, H. K., Marques, M. D., McLean, S. A., Slater, A., & Paxton, S. J. (2021). Social media, body satisfaction and well-being among adolescents: A mediation model of appearance-ideal internalization and comparison. Body Image, 36, 139-148.

 Noon, E. J., Schuck, L. A., Guțu, S. M., Şahin, B., Vujović, B., & Aydın, Z. (2021). To compare, or not to compare? Age moderates the relationship between social comparisons on instagram and identity processes during adolescence and emerging adulthood. Journal of Adolescence, 93, 134-145.

**Reviewers:**

**Ethics:**

**It would be interesting to get more details about the consent form obtention procedure, as well as general ethics issues. For example, it is said that “those aged 16 to 18 answered the children themselves but with the consent of the parents, which were present while they answered as the legislation indicates”.  How could the authors be sure of this statement?**

**Response:**

 The panel was contracted to the company ODEC. This company has worked with (Dynata) a software that complies with all legal issues in relation to data protection and response protocols. Those under 16 years of age answered with their parents by their side and those aged 16 to 18 answered by themselves with the consent of their parents present while answering as required by law.

 ODEC offers digital solutions specialized in market research, statistics, media and marketing.
